# Using immunovascular characteristics to predict very early recurrence and prognosis of resectable intrahepatic cholangiocarcinoma

**DOI:** 10.1186/s12885-023-11476-z

**Published:** 2023-10-19

**Authors:** Ying Xu, Zhuo Li, Yanzhao Zhou, Yi Yang, Jingzhong Ouyang, Lu Li, Zhen Huang, Feng Ye, Jianming Ying, Hong Zhao, Jinxue Zhou, Xinming Zhao

**Affiliations:** 1 Department of Diagnostic Radiology, National Cancer Center/National Clinical Research Center for Cancer/Cancer Hospital, Chinese Academy of Medical Sciences and Peking Union Medical College, Beijing, China; 2 Department of Pathology, National Cancer Center/National Clinical Research Center for Cancer/Cancer Hospital, Chinese Academy of Medical Sciences and Peking Union Medical College, Beijing, China; 3 Department of Hepatobiliary and Pancreatic Surgery, The Affiliated Cancer Hospital of Zhengzhou University & Henan Cancer Hospital, Zhengzhou, Henan China; 4 Department of Hepatobiliary Surgery, National Cancer Center/National Clinical Research Center for Cancer/Cancer Hospital, Chinese Academy of Medical Sciences and Peking Union Medical College, Beijing, China; 5 Key Laboratory of Gene Editing Screening and Research and Development (R&D) of Digestive System Tumor Drugs, Chinese Academy of Medical Sciences and Peking Union Medical College, Beijing, China

**Keywords:** Cholangiocarcinoma, Tertiary lymphoid structures, Microvascular invasion, Recurrence, Prognosis

## Abstract

**Objective:**

To predict the very early recurrence (VER) of patients with intrahepatic cholangiocarcinoma (ICC) based on TLSs and MVI status, and further perform prognosis stratifications.

**Methods:**

A total of 160, 51 ICC patients from two institutions between May 2012 and July 2022 were retrospectively included as training, external validation cohort. Clinical, radiological and pathological variables were evaluated and collected. Univariate and multivariate analysis were applied to select the significant factors related to VER of ICC. The factors selected were combined to perform stratification of overall survival (OS) using the Kaplan-Meier method with the log-rank test.

**Results:**

Overall, 39 patients (24.4%) had VER, whereas 121 (75.6%) did not (non-VER group). In the training cohort, the median OS was 40.5 months (95% CIs: 33.2–47.7 months). The VER group showed significantly worse OS than the non-VER group (median OS: 14.8, 95% CI:11.6–18.0 months vs. 53.4, 34.3–72.6 months; *p*<0.001), and it was confirmed in the validation cohort (median OS: 22.1, 95% CI: 8.8–35.4 months vs. 40.1, 21.2–59.0 months; *p* = 0.003). According to the univariate analysis, four variables were significantly different between the VER group and non-VER group (TLSs status, *p* = 0.028; differentiation, *p* = 0.023; MVI status, *p* = 0.012; diameter, *p* = 0.028). According to the multivariate analysis, MVI-positive status was independently associated with a higher probability of VER (odds ratio [OR], 2.5; 95% CIs,1.16–5.18; *p* = 0.018), whereas intra-tumoral TLSs-positive status was associated with lower odds of VER (OR, 0.43; 95% CIs, 0.19–0.97; *p* = 0.041). Based on the TLSs and MVI status, patients of ICC were categorized into four groups: TLSs-positive and MVI-negative (TP/MN); TLSs-negative and MVI-negative (TN/MN); TLSs-positive and MVI-positive (TP/MP), TLSs-negative and MVI-positive groups (TN/MP). In the training cohort, the four groups could be correlated with OS significantly (*p*<0.001), and it was confirmed in the validation cohort (*p*<0.001).

**Conclusion:**

Intra-tumoral TLSs and MVI status are independent predictive factors of VER after surgery, based on which immunovascular stratifications are constructed and associated with OS significantly of resectable intrahepatic cholangiocarcinoma.

**Supplementary Information:**

The online version contains supplementary material available at 10.1186/s12885-023-11476-z.

## Introduction

Intrahepatic cholangiocarcinoma (ICC) is the second most common primary liver malignancy (10-15%) after hepatocellular carcinoma (HCC), with a globally increasing incidence and mortality [1, 2]. ICC has a more aggressive biological behavior compared with HCC [3], and surgical resection provides the best option of potential cure for resectable ICC. However, 50–70% of patients will experience tumor recurrence after surgery, which limit long-term survival of patient with ICC [3–5].

Cholangiocarcinoma (CCA) has abundant desmoplastic stroma with tumor structures infiltration and a rich tumor microenvironment (TME) [6]. The tumor microenvironment (TME) plays an important role in progression and metastases of ICC [7]. TME composes of endothelial cells, immune cells, cancer-associated fibroblasts (CAFs), and extracellular matrices (ECMs), which are correlated with prognosis and immune response [8]. Recently, tumor-associated tertiary lymphoid structures (TLSs), ectopic aggregates of immune cells with similarities to secondary lymphoid organs (SLO), have attracted extensive attention owing to its potential prognostic value and guiding significance of immunotherapy [9]. Ding et al. reported that intra-tumor region TLSs of ICC were positively correlated with favorable prognosis whereas peri-tumor region signified worse survival [10]. Microvascular invasion (MVI) is defined as endovascular cancer cell nests found under microscopic examination, which mainly located at tumor-adjacent hepatic vein and portal vein [9]. MVI has been reported as an independent risk factor for both worse recurrence free survival (RFS) and overall survival (OS) of ICC [11–13]. Knowing the MVI status would facilitate the adoption of more active treatment methods for high-risk patients, such as anatomical resection to expand the distance of the surgical margin and adjuvant therapies, including TACE, radiotherapy, and immunotherapy, to achieve a better prognosis [11].

Remarkably, previous studies reported that approximately one-quarter patients with ICC experienced tumor recurrence within 6 months after initial resection, which was defined as very early recurrence (VER) [3, 4]. Patients with VER after surgery had worse OS than those without VER [4]. Identifying risk factors of VER is conducive to postoperative surveillance and subsequent precise adjuvant therapeutic strategy. Previous study indicated that age, race, MVI and tumor staging characteristics were risk factors of patients with VER [4].

Kurebayashi et al. identified four distinct immunovascular subtypes of HCC correlated with different prognosis, which revealed detailed relationship and reciprocal interaction between tumor vessels and immune cells [14]. To our knowledge, no previous study has explored the relationship between characteristics of TME and VER of ICC. We hypothesized that integrating characteristics of aggressive behavior (MVI) and TME (TLSs) could effectively predict VER of ICC. Therefore, the purpose of this study was to identify the independent predictive factors of VER and develop a prognosis stratification tool correlated with OS for patients with ICC after surgery, which will help oncologists make postoperative therapeutic decisions.

## Materials and methods

### Patients

**Fig. 1:**
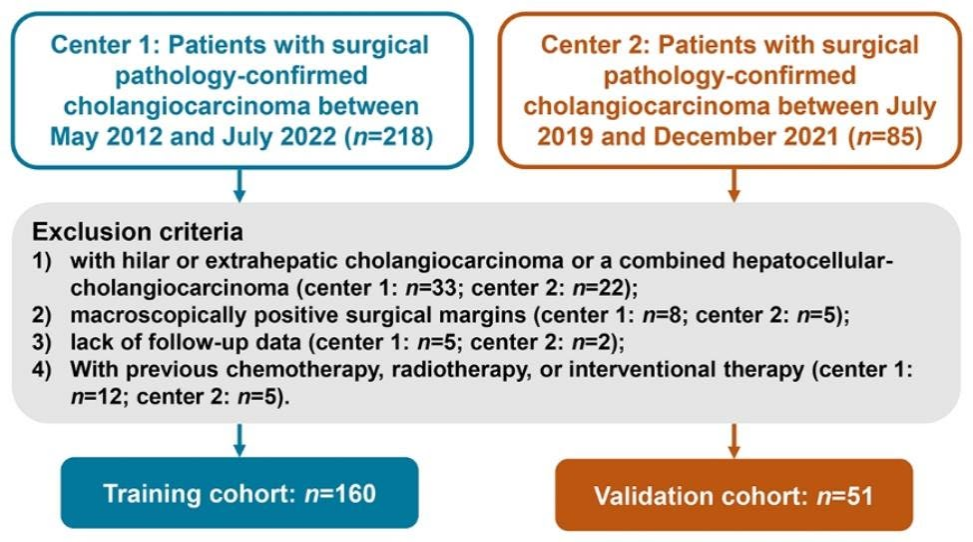
Flow Chart of patient selection

Patients with surgical pathology-confirmed cholangiocarcinoma were retrospectively included. This study included two independent ICC cohorts: Two hundred and eighteen patients as training cohort from Cancer Hospital, Chinese Academy of Medical Sciences (between May 2012 and July 2022), and 85 patients as external validation cohort from Affiliated Cancer Hospital of Zhengzhou University (between July 2019 and December 2021). The inclusion criteria were as follows: (1) patients with surgical pathology-confirmed ICC; (2) patients without previous treatment for ICC; and (3) patients with complete preoperative clinical, radiological data within 1 month before surgery and complete postoperative pathologic data. The exclusion criteria were as follows: (1) patients with hilar or extrahepatic cholangiocarcinoma or a combined hepatocellular-cholangiocarcinoma; (2) macroscopically positive surgical margins; (3) lack of follow-up data; (4) previous treatment for liver lesions (chemotherapy, radiotherapy, or interventional therapy). A flowchart of the patient selection process is shown in Fig. 1. This study was approved by the institutional review board of each hospital, and the requirement for patient informed consent was waived for this retrospective analysis. All the clinical, radiological and pathological data of the patients was anonymized or de-identified.

### Preoperative clinical, radiological variables

Demographic, clinical variables potentially associated with the postoperative prognosis of ICC were collected from electronic medical record [15–18], including age, sex, presence of hepatitis B virus, liver cirrhosis, fatty liver, preoperative serum levels of carbohydrate antigen 19-9 (CA19-9), alanine aminotransferase (ALT), aspartate transaminase (AST), total bilirubin (TBil), albumin (ALB).

Radiological evaluation was performed by two radiologists (Radiologist 1, L.L. M.D., and Radiologist 2, Y.X. M.D., with ten and six years of experience in abdominal radiology, respectively) independently on the preoperative MRI scans. Any discrepancy between the two radiologists was adjudicated by a third senior radiologist (Radiologist 3, F.Y., M.D. with 18 years of experience in abdominal radiology) to reach a consensus among the three radiologists. All three radiologists were blinded to the patients’ clinical data and pathological results. For patients with multiple lesions, the largest lesion was selected for evaluation. The MRI findings of each lesion were evaluated as follows: tumor location, tumor size and number, morphology, border, satellite nodules, intra-tumor vessels, peri-tumor biliary dilatation, and hepatic capsule retraction. Moreover, the signal features of T1WI (in- and out-phase), T2WI, DWI, and contrast-enhanced scans were evaluated. Details of the MRI findings and signal features were presented in **S-Table 1**.

### Postoperative and histopathologic analysis

Pathologic variables collected from electronic medical record included macrovascular invasion, differentiation, nerve invasion, microvascular invasion, intra-tumoral necrosis (necrotic area > 5%), and portal thrombus. Macrovascular invasion was defined as invasion of the hepatic artery, portal vein, or hepatic veins. Microvascular invasion was defined as presence of a tumor cell nest in the small portal vein, hepatic vein or large capsular vessel covered with endothelial cell under microscopic examination [19]. The type of liver surgery, adjuvant therapy performed were recorded. Major hepatectomy was defined as resection of 3 or more Couinaud segments [20].

Intra-tumoral and peri-tumoral TLSs status were assessed through reviewing the pathological hematein-eosinsaffron stained slides of each lesion for whole slide images (WSIs) by two pathologists (Z.L.MD with 10-year and J.M.Y.MD with 20-year experience in cancer pathology). Both the pathologists were blinded to the patients’ clinical data and radiological results. Any discrepancy between the two pathologists was discussed to reach a consensus. The existence of TLSs was assessed morphologically as described previously [10, 21, 22]. Briefly, TLSs were classified as 3 categories according to their maturation stages: (1) lymphoid aggregates (Agg): vague, ill-defined clusters of lymphocytes; (2) primary lymphoid follicles (Fol-I): lymphoid follicles without germinal center formation and (3) secondary lymphoid follicles (Fol-II): lymphoid follicles with germinal center formation. TLSs-negative was defined as tumors without any TLSs and TLSs-positive was defined as tumors with at least one TLS.

### Follow-up

Regular follow-up was conducted every three months until 2 years after surgery, twice per year in the third, fourth, and fifth year, and once a year after that. Disease recurrence was confirmed by CT, MRI, or PET-CT. RFS was defined as the date from the surgery to disease recurrence, or the last follow-up date. OS was the time from the date of surgery to death by any cause or the last follow-up date. The last follow up was conducted on November 27th, 2022. The VER of ICC was defined as the incidence of recurrence within 6 months after resection based on previous studies [4, 23].

### Statistical analysis

The univariate analysis was performed, and variables with *p*-values of < 0.05 were applied to a multivariate logistic regression analysis. Odds ratios (OR) as estimates of relative risk with 95% confidence intervals (CIs) were calculated for each independent factor. The factors selected were combined to perform prognosis stratification. Differences in OS between different groups were assessed using the Kaplan-Meier method with the log-rank test. Statistical analyses were performed with SPSS (version 25.0; IBM), R statistical software (version 3.3.3; https://www.r-project.org). The chi-square test or Fisher’s exact test was used for categorical variables, and the Mann–Whitney U test or Student’s t-test was used for continuous variables. P < 0.05 was considered statistically significant.

## Results

### Baseline characteristics

Based on the inclusion and exclusion criteria, 160 patients from our institution between June 2015 and July 2022 were included as training cohort. Another cohort of 51 patients with ICC between July 2016 and December 2021 was collected from another medical center based on the same criteria as the external validation cohort. The comparisons of clinical, laboratory, and pathological variables between the training and validation cohorts were summarised in Table 1. Age of patients in training and validation cohorts were 58.62 ± 9.15 and 56.61 ± 9.37. Ninety-two (57.5%) and 31 (60.8%) males were included in the training and validation cohorts, respectively. All the baseline variables between the two cohorts have no statistical differences.

**Table 1:** The baseline characteristics of patients included in the training and validation cohorts

Characteristics	Training cohort n = 160	Validation cohort n = 51	P value
**Preoperative variables**			
Age (mean ± SD)	58.619 ± 9.149	56.608 ± 9.368	0.176
Sex, male n (%)	92 (57.5%)	31 (60.8%)	0.679
ALT > 40U/L, n (%)	13 (8.1%)	5 (9.8%)	0.932
AST > 40U/L, n (%)	13 (8.1%)	7 (13.7%)	0.360
TBil > 20.4mmol/L, n (%)	9 (5.6%)	2 (3.9%)	0.909
ALB < 35 g/L, n (%)	3 (1.9%)	1 (2.0%)	1.000
CA199 > 37U/ml, n (%)	90 (56.3%)	27 (52.9%)	0.679
HBV positive, n (%)	92 (57.5%)	26 (51.0%)	0.414
Liver cirrhosis, n (%)	86 (53.8%)	25 (49.0%)	0.556
Liver steatosis, n (%)	38 (23.8%)	14 (27.5%)	0.593
Location, n (%)			0.510
right lobe	70 (43.8%)	25 (49.0%)	
left lobe	90 (56.2%)	26 (51.0%)	
Subcapsular, n (%)	111 (69.4%)	32 (62.7%)	0.378
Satellite nodules, n (%)	15 (9.4%)	2 (3.9%)	0.342
Diameter, median (IQR)	4.8 (3.60, 6.30)	4.9 (3.75, 6.80)	0.384
Number, n (%)			0.227
1	152 (95.0%)	51 (100.0%)	
>1	8 (5.0%)	0 (0.0%)	
Intratumor hemorrhage, n (%)	6 (3.8%)	3 (5.9%)	0.796
Biliary dilatation, n (%)	44 (27.5%)	16 (31.4%)	0.593
Hepatic capsule retraction, n (%)	108 (67.5%)	34 (66.7%)	0.912
Regular morphology, n (%)	14 (8.8%)	1 (2.0%)	0.183
Well-defined border, n (%)	142 (88.8%)	42 (82.4%)	0.234
T2WI signal, n (%)			
central high	57 (35.6%)	20 (39.2%)	0.643
homogeneous high	73 (45.6%)	24 (47.1%)	0.858
peripheral rim high	30 (18.8%)	7 (13.7%)	0.411
Arterial enhancement pattern, n (%)			0.877
peripheral rim enhancement	88 (55%)	28 (54.9%)	
diffuse hypoenhancement	45 (28.1%)	13 (25.5%)	
diffuse hyperenhancement	27 (16.9%)	10 (19.6%)	
Centripetal enhancement, n (%)	89 (55.6%)	30 (58.8%)	0.688
Wash in and wash out, n (%)	18 (11.2%)	7 (13.7%)	0.634
Persistent enhancement, n (%)	31 (19.4%)	7 (13.7%)	0.361
Intratumor vessels, n (%)	18 (11.2%)	6 (11.8%)	0.920
Peritumoral arterial enhancement, n (%)	61 (38.1%)	20 (39.2%)	0.889
DWI rim high signal, n (%)	66 (41.2%)	22 (43.1%)	0.812
**Postoperative variables**			
Macrovascular invasion, n (%)	31 (19.4%)	8 (15.7%)	0.555
Lymph node metastasis, n (%)	46 (28.8%)	16 (31.4%)	0.739
AJCC 8th TNM stage, n (%)			0.834
1	77 (48.1%)	23 (45.1%)	
3	56 (35.0%)	21 (41.2%)	
2	26 (16.3%)	7 (13.7%)	
4	1 (0.6%)	0 (0.0%)	
Intra-tumoral TLSs-positive, n (%)	65 (40.6%)	23 (45.1%)	0.573
Peri-tumoral TLSs-positive, n (%)	49 (30.6%)	20 (39.2%)	0.255
Differentiation, n (%)			0.812
well or moderate	66 (41.2%)	22 (43.1%)	
poor	94 (58.8%)	29 (56.9%)	
Nerve invasion, n (%)	51 (31.9%)	16 (31.4%)	0.946
MVI-positive, n (%)	63 (39.4%)	23 (45.1%)	0.469
Necrosis, n (%)	139 (86.9%)	45 (88.2%)	0.800
Type of liver surgery, n (%)			0.565
major resection	54 (33.7%)	15 (29.4%)	
minor resection	106 (66.3%)	36 (70.6%)	
Adjuvant therapy performed, n (%)	53 (33.1%)	21 (41.2%)	0.294
VER, n (%)	39 (24.4%)	11 (21.6%)	0.681
Recurrence, n (%)	99 (61.9%)	35 (68.6%)	0.383
RFS, median (IQR)	10.33 (6.53, 36.58)	9.5 (6.87, 27.00)	0.520
Death, n (%)	94 (58.8%)	35 (68.6%)	0.208
OS, median (IQR)	35.68 (16.27, 53.97)	26.67 (15.22, 44.23)	0.194

Notes: ALT, alanine aminotransferase; AST, aspartate transaminase; TBil, total bilirubin; ALB, albumin; CA199, carbohydrate antigen 199; HBV, hepatitis B virus; TLSs, tertiary lymphoid structures; MVI, microvascular invasion; VER, very early recurrence; RFS, recurrence-free survival; OS, overall survival

### Correlations of VER with OS

**Fig. 2:**
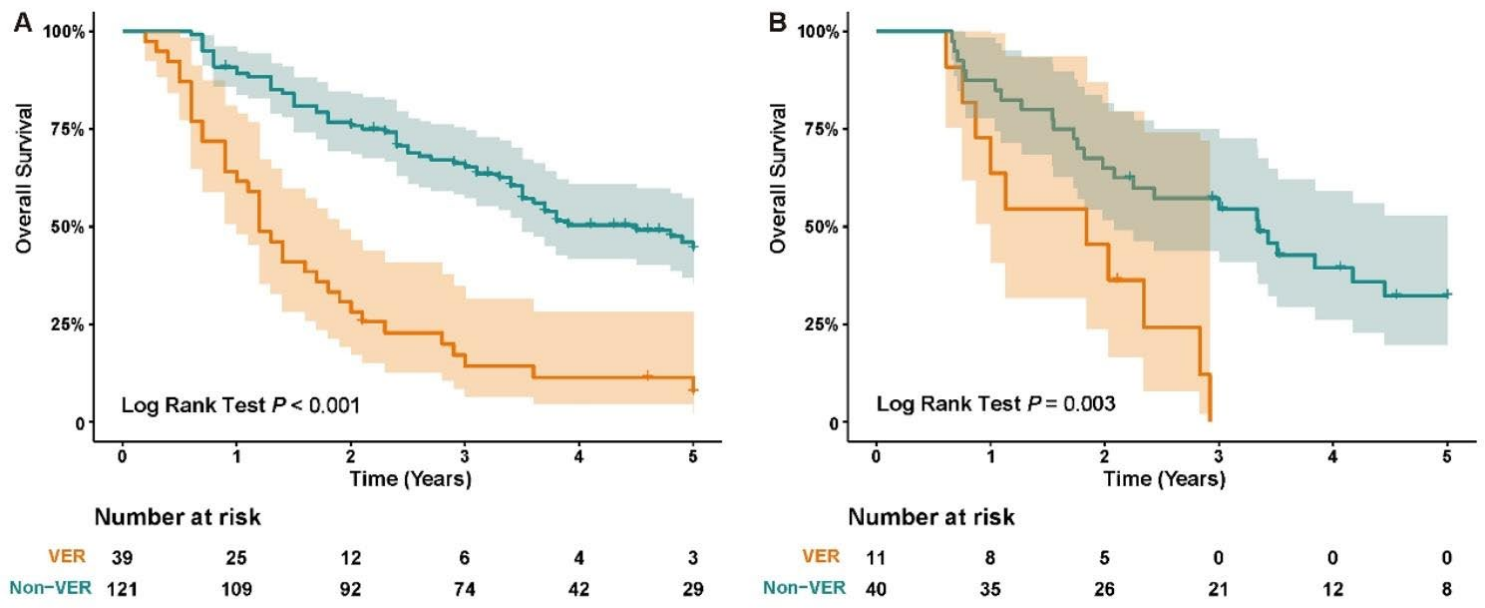
Kaplan-Meier curves for OS of patients with ICC in training cohort **(A)** and validation cohort **(B)** as categorized by VER and non-VER.

In the training cohort, ninety-nine patients (61.9%) experienced recurrence, and ninety-four (58.8%) patients died during a median follow-up duration of 60.8 months (95% confidence intervals [CIs]: 58.0–63.6 months). The median OS was 40.5 months (95% CIs: 33.2–47.7 months), and the median RFS was 14.1 months (95% CIs: 6.3–21.9 months). The VER group showed significantly worse OS than the non-VER group (median OS: 14.8, 95% CI:11.6–18.0 months vs. 53.4, 34.3–72.6 months; *p* < 0.001; Fig. 2A). The 6-, 12-, 24-, 36-, 48-, and 60-month survival rates of the VER and non-VER groups were shown as **S-Fig. 1**.

In the validation cohort, thirty-five patients (68.6%) experienced recurrence, and 35 (68.6%) patients died during a median follow-up duration of 60.0 months (95% CIs: 58.7–61.3 months). The median OS was 28.1 months (95% CIs: 16.8–39.4 months), and the median RFS was 10.7 months (95% CIs: 6.6–14.7 months). The VER group showed significantly worse OS than the non-VER group (median OS: 22.1, 95% CI: 8.8–35.4 months vs. 40.1, 21.2–59.0 months; *p* = 0.003; Fig. 2B).

### Factors selection for prediction of VER

Overall, 39 patients (24.4%) had VER, whereas 121 (75.6%) did not (non-VER group); in the non-VER group, 60 patients (37.5%) had a recurrence more than 6 months after resection and 61 patients (38.1%) didn’t experience tumor recurrence during the follow-up period.

According to the univariate analysis, four variables were significantly different between the VER group and non-VER group (TLSs status, *p* = 0.028; differentiation, *p* = 0.023; MVI status, *p* = 0.012; diameter, *p* = 0.028) (Table 2). According to the multivariate analysis, MVI-positive and intra-tumoral TLSs-positive status were independent predictive factors of VER. MVI-positive was independently associated with a higher probability of VER (odds ratio [OR], 2.5; 95% CIs,1.16–5.18; *p* = 0.018), whereas intra-tumoral TLSs-positive status was independently associated with lower odds of VER (OR, 0.43; 95% CIs, 0.19–0.97; *p* = 0.041). (Table 2).

**Table 2:** Univariate and multivariate analysis of the preoperative clinical, radiologic, and postoperative pathological variables between VER and non-VER groups in the training cohort

		Univariate analysis				Multivariate analysis	
Characteristics		non-VER (n = 121)	VER (n = 39)	*p* value		OR (95% CI)	*p* value
**Preoperative variables**							
Age (mean ± SD)		59.041 ± 9.292	57.308 ± 8.673	0.305			
Sex, male n (%)		68 (56.2%)	24 (61.5%)	0.557			
ALT > 40U/L, n (%)		9 (7.4%)	4 (10.3%)	0.823			
AST > 40U/L, n (%)		11 (9.1%)	2 (5.1%)	0.652			
TBil > 20.4mmol/L, n (%)		6 (5.0%)	3 (7.7%)	0.807			
ALB < 35 g/L, n (%)		3 (2.5%)	0 (0%)	0.754			
CA199 > 37U/ml, n (%)		66 (54.5%)	24 (61.5%)	0.444			
HBV positive, n (%)		72 (59.5%)	20 (51.3%)	0.366			
Liver cirrhosis, n (%)		67 (55.4%)	19 (48.7%)	0.469			
Liver steatosis, n (%)		29 (24.0%)	9 (23.1%)	0.910			
Location, n (%)				0.132			
right lobe		57 (47.1%)	13 (33.3%)				
left lobe		64 (52.9%)	26 (66.7%)				
Subcapsular, n (%)		82 (67.8%)	29 (74.4%)	0.437			
Satellite nodules, n (%)		9 (7.4%)	6 (15.4%)	0.244			
Diameter, median (IQR)		4.5 (3.60, 5.90)	5.8 (4.20, 6.85)	0.028^*^			0.170
Number, n (%)				0.642			
1		116 (95.9%)	36 (92.3%)				
>1		5 (4.1%)	3 (7.7%)				
Intratumor hemorrhage, n (%)		4 (3.3%)	2 (5.1%)	0.971			
Regular morphology, n (%)		13 (10.7%)	1 (2.6%)	0.213			
Well-defined border, n (%)		106 (87.6%)	36 (92.3%)	0.605			
T2WI signal, n (%)				0.253			
central high		43 (35.5%)	14 (35.9%)				
homogeneous high		52 (43%)	21 (53.8%)				
peripheral rim high		26 (21.5%)	4 (10.3%)				
Arterial enhancement pattern, n (%)				0.138			
peripheral rim enhancement		62 (51.3%)	26 (66.7%)				
diffuse hypoenhancement		35 (28.9%)	10 (25.6%)				
diffuse hyperenhancement		24 (19.8%)	3 (7.7%)				
Centripetal enhancement, n (%)		67 (55.4%)	22 (56.4%)	0.910			
Wash in and wash out, n (%)		17 (14.0%)	1 (2.6%)	0.092			
Persistent enhancement, n (%)		20 (16.5%)	11 (28.2%)	0.109			
Intratumor vessels, n (%)		14 (11.6%)	4 (10.3%)	1.000			
Peritumoral arterial enhancement, n (%)		42 (34.7%)	19 (48.7%)	0.117			
DWI rim high signal, n (%)		50 (41.3%)	16 (41.0%)	0.974			
Peri-tumor biliary dilatation		116 (72.5%)	44 (27.5%)	0.179			
Hepatic capsule retraction		52 (32.5%)	108 (67.5%)	0.295			
**Postoperative variables**							
Intra-tumoral TLSs-positive, n (%)		55 (45.5%)	10 (25.6%)	0.028^*^		0.428 (0.189–0.967) (positive vs. negative)	0.041^*^
Peri-tumoral TLSs-positive, n (%)		41 (33.9%)	8 (20.5%)	0.115			
Macrovascular invasion, n (%)		26 (21.5%)	5 (12.8%)	0.234			
Differentiation, n (%)				0.023^*^			0.077
well or moderate		56 (46.3%)	10 (25.6%)				
poor		65 (53.7%)	29 (74.4%)				
Nerve invasion, n (%)		35 (28.9%)	16 (41.0%)	0.158			
MVI-positive, n (%)		41 (33.9%)	22 (56.4%)	0.012^*^		2.454 (1.163–5.180) (positive vs. negative)	0.018^*^
Necrosis, n (%)		105 (86.8%)	34 (87.2%)	0.948			
Portal thrombus, n (%)		16 (13.2%)	4 (10.3%)	0.835			
Adjuvant therapy performed, n (%)		37 (31.6%)	16 (41.0%)	0.283			

Notes: ALT, alanine aminotransferase; AST, aspartate transaminase; TBil, total bilirubin; ALB, albumin; CA199, carbohydrate antigen 199; HBV, hepatitis B virus; TLSs, tertiary lymphoid structures; MVI, microvascular invasion     * Statistically significant

**Fig. 3:**
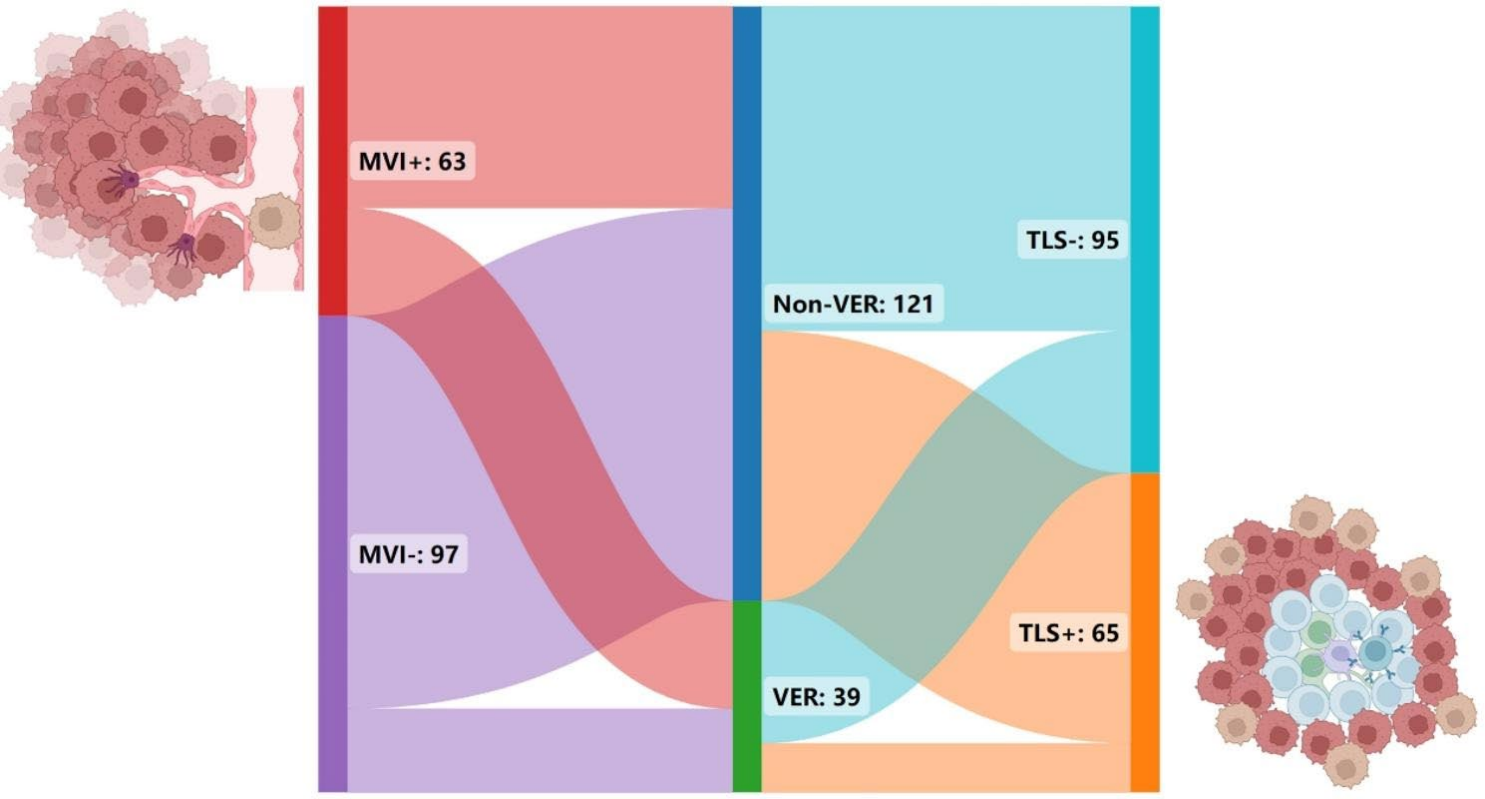
The Sankey diagram represents the different proportions of MVI (left), VER (middle), TLSs (right) status. In the 160 patients, 63 patients were MVI-positive (red bar) and 97 patients were MVI-negative (purple bar); 121 patients were non-VER (deep blue bar) and 39 patients were VER (green bar); 95 patients were TLSs-negative (light blue bar) and 65 patients were TLSs-positive (orange bar). The strips (lines) connecting different bars represent branches (shunts), and the width of the branches corresponds to the size of the data flow. For example, the red strips (lines) connecting the MVI and VER bars represents that in the 63 MVI-positive groups, 22 patients were VER (narrow strip) and 41 patients were non-VER (wide strip)

Sixty-three (39.4%) patients were MVI-positive while 97 (60.6%) patients were MVI-negative. Sixty-five (40.6%) patients were TLSs-positive while 95 (59.4%) patients were TLSs-negative. Twenty-two (56.4%), 41 (33.9%) patients were MVI-positive in VER group, non-VER group, respectively. Ten (25.6%), 55 (45.5%) patients were TLSs-positive in VER group, non-VER group, respectively (Fig. 3). According to the TLSs and MVI status, patients of ICC were categorized into four groups: TLSs-positive and MVI-negative (TP/MN); TLSs-negative and MVI-negative (TN/MN); TLSs-positive and MVI-positive (TP/MP), TLSs-negative and MVI-positive groups (TN/MP).

### Correlations of immunovascular stratifications with OS

**Fig. 4:**
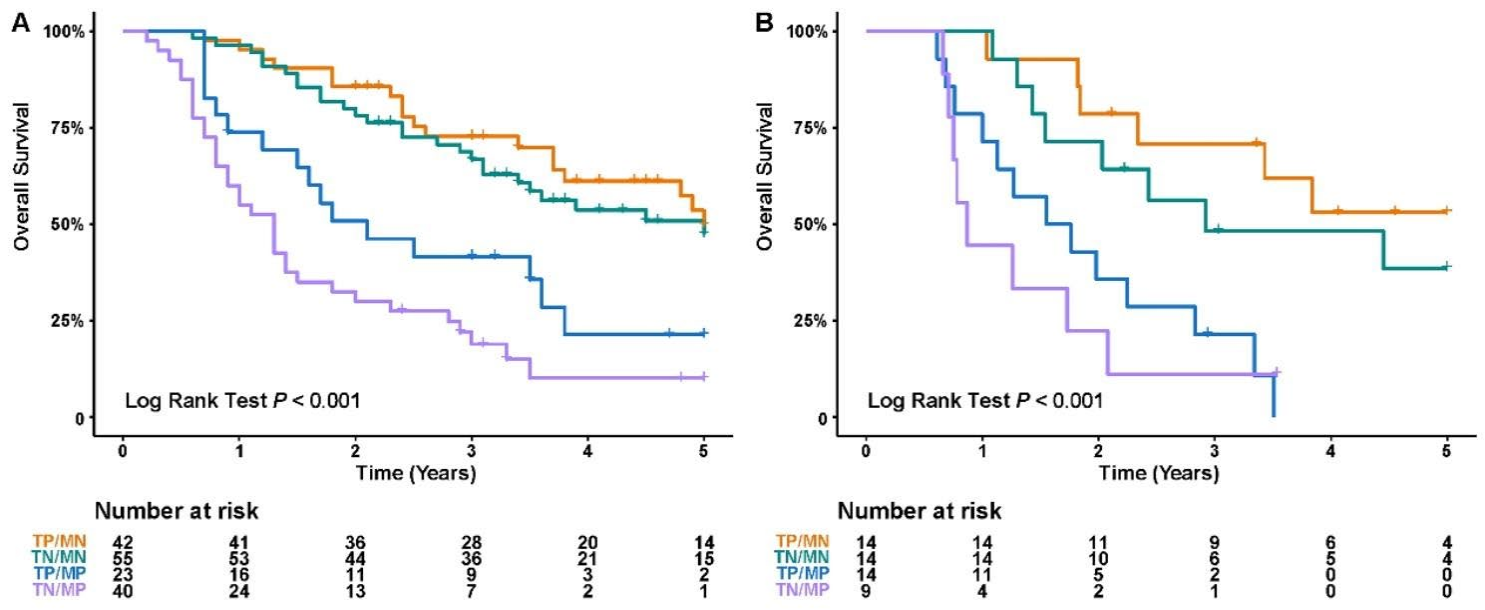
Kaplan-Meier curves for OS of 160 patients in the training cohort (A), for OS of 51 patients in the validation cohort (B) with ICC as categorized by the TP/MN, TN/MN, TP/MP, TN/MP groups were conducted

In the training cohort, forty-two (26.2%), 55 (34.4%), 23(14.4%), 40 (25%) patients were stratified into TP/MN, TN/MN, TP/MP, TN/MP groups. And the four groups could be correlated with OS significantly (*p* < 0.001, Fig. 4A). The 6-, 24-, and 60-month survival rates were 100.0%, 85.7%, 59.5% in TP/MN group, 100.0%, 80%, 54.5% in TN/MN group, 100%, 52.2%, 30.4% in TP/MP group, and 87.5%, 30%, 15% in TN/MP group, respectively.

In the validation cohort, fourteen (27.5%), 14 (27.5%), 14(27.5%), 9 (17.5%) patients were stratified into TP/MN, TN/MN, TP/MP, TN/MP groups. And the four groups could be correlated with OS significantly (*p* < 0.001, Fig. 4B).

## Discussion

Tsilimigras et al. noticed that using a cutoff of 2 years for early recurrence of ICC may be problematic because many patients with ICC have recurrence much earlier within the very first months following resection [4]. In their study there was 22.3% patients developed recurrence within 6months after resection and in this study 24.4% patients had VER. In our study, we demonstrated that VER after resection was a risk factor of poor survival and VER group showed significantly worse OS than the non-VER group, which is consistent with the previous study [4]. The multivariate analysis results indicated that intra-tumoral TLSs and MVI status were independent predictors of VER for ICC. We attempted to predict OS by combination of TLSs and MVI status, based on which patients of ICC were categorized into four groups: TP/MN, TN/MN, TP/MP, and TN/MP groups. The four groups could be correlated with OS significantly and it was confirmed in validation cohort.

Tumor size and number (e.g., tumor burden), MVI, lymph node metastasis, poor/undifferentiated tumor grade have been associated with risk of recurrence among patients with ICC in previous studies [5, 24, 25], but none of the studies reported the relationship between VER and TLSs status. In our study the tumor size and differentiation were found significant only in the univariate analysis. Our study demonstrated that ICC of VER group were more frequently MVI-positive and TLSs-negative than those of non-VER group. The four groups categorized by immunovascular characteristics were correlated with OS significantly, and the median OS proved to be best in the TP/MN group, worst in the TN/MP groups.

Chemotherapy is recommended as postoperative adjuvant therapy of resectable ICC [26]. Chemotherapy combined with immunotherapy are recommended as first-line systematic therapy for advanced biliary tract cancer [27]. Chemotherapy plus antiangiogenic therapy plus immunotherapy for advanced biliary tract cancer are now being exploring and showed promising clinical benefit [28, 29]. Some implications from systematic therapy may be brought into adjuvant therapy.

The composition of TLSs included CD20 + B cells, CD3 + T cells, CD4 + T follicular helper (TFH) cells, CD8 + cytotoxic T cells, CD4 + T helper 1 (TH1) cells, regulatory T cells (Tregs) and CD21 + follicular dendritic cells (FDCs) [30–33], which is defined as immune infiltrates in tumors. It indicated a better response to immunotherapy independent of PD-L1 expression status and CD8 + T cell density, not only for ICC, but also other types of solid tumors including HCC, melanoma, et.al [34–36]. For patients with TLSs-positive ICC, chemotherapy plus immunotherapy might be considered as adjuvant therapy.

MVI has been reported associated with poor RFS and OS of ICC [11]. In the present study, it is also identified as an independent predictive factor of VER. For patients with MVI-positive HCC, adjuvant transarterial chemoembolization (TACE) or hepatic arterial infusion chemotherapy (HAIC) could significantly reduce rates of recurrence and prolong survival [37–39]. Peng et al. reported that MVI was a predictor of therapy efficacy of Sorafenib and TACE for recurrent HCC [40]. We speculated that using chemotherapy plus antiangiogenic therapy plus local therapy as adjuvant therapy might bring survival benefit for patients with MVI-positive ICC. As a result, the immunovascular characteristics is beneficial to accurately guide postoperative adjuvant therapy for ICC patients, especially for TP and MP groups.

There are some limitations in this study. First, this is a retrospective study and selection bias is inevitable. Second, the sample size is limited and the further study with larger sample size was needed. Third, no preoperative factors were selected in the multivariate and this may be attributed to sample size bias.

In conclusion, intra-tumoral TLSs and MVI status are independent predictive factors of VER after surgery, based on which immunovascular stratifications are constructed and associated with OS significantly of resectable ICC. The immunovascular characteristics might be helpful to ICC patients for personalized therapy.

### Electronic supplementary material

Below is the link to the electronic supplementary material.

Supplementary Material 1

Supplementary Material 2

## Data Availability

The datasets used or analyzed during the current study are available from the corresponding author on reasonable request. Requests to access these datasets could be directed to dr_fengye_ncc@163.com.
