# Supplementary material for: Using immunovascular characteristics to predict very early recurrence and prognosis of resectable intrahepatic cholangiocarcinoma
Source: BMC Cancer. 2023 Oct 19;23:1009. doi: 10.1186/s12885-023-11476-z (PMC10588260; doi:10.1186/s12885-023-11476-z)
Supplement: Supplementary file 1 — Supplementary Material 1 [file 12885_2023_11476_MOESM1_ESM.docx]

S-Table 1. The MRI findings and signal characteristics.

| MRI findings and signal characteristics | Definition[1，2，3，4，5，6，7] |
| --- | --- |
| T1WI in- and out-phase |  |
| Hemorrhagic component | Amorphous areas of high signal on T1WI in-phase which do not lose signal on out-phase or fat-suppressed image. |
| T2WI |  |
| Homogeneous high signal | Homogeneous slightly higher, medium higher, higher signal than liver parenchyma on T2WI. |
| Peripheral rim high signal | Peripheral ring-like hyperintense with  central hypointense areas. |
| Central high signal | Central hyperintense areas with peripheral ring-like hypointense. |
| DWI |  |
| Rim high signal | Peripheral ring-like hyperintense with  central hypointense areas on DWI. |
| Arterial phase |  |
| Diffuse hyperenhancement | Homogeneous higher intensity than liver parenchyma in >70% of the tumor area on arterial phase enhancement. |
| Peripheral rim hyperenhancement | Peripheral ring-like hyperintense in 10%-70% tumor area with central hypointense areas on arterial phase enhancement. |
| Diffuse hypoenhancement | Homogeneous lower intensity than liver parenchyma on arterial phase enhancement. |
| Peritumoral enhancement | Gross hyperenhancement outside the tumor border in the arterial phase, becoming isointense in later dynamic phases compared with the background liver parenchyma. |
| Enhancement pattern |  |
| Wash in and wash out | Arterial hyperenhancement with isointense or hypointense in the portal venous and delayed phase. |
| Centripetal enhancement | Rim or peripheral arterial-phase enhancement, and centripetal enhancement on the delayed phase. |
| Persistent enhancement | Hyperenhancement on the arterial phase and persistent enhancement in the portal venous and delayed phase. |
| Tumor location |  |
| Left or right lobe | Left lobe consists of 2-4 segments of liver and right lobe consists of 5-8 segments. |
| Subcapsular | The lesion is adjacent to the liver capsule on the images. |
| Tumor size | Measurement of the maximum diameter in the delayed phase on axial MRI. |
| Satellite nodules | Distinct tumor nodules in the vicinity of the main tumor. |
| Regular morphology | Tumor without a budding portion protruding into the liver parenchyma. |
| Clear border | Tumor had smooth margins and the boundary with the surrounding liver parenchyma was clear at each phase. |
| Intratumor vessels | Blood vessels including hepatic arteries, hepatic veins, portal veins, and their branches go through the tumor. |
| Hepatic capsule retraction | Invagination or focal flattening of the typical smooth contour of the liver capsule. |

Note: WI= weighted image.

1 Renzulli M, Brocchi S, Cucchetti A et al (2016) Can Current Preoperative Imaging Be Used to Detect Microvascular Invasion of Hepatocellular Carcinoma? Radiology 279:432-442

2 Min JH, Kim YK, Choi SY et al (2019) Intrahepatic Mass-forming Cholangiocarcinoma: Arterial Enhancement Patterns at MRI and Prognosis. Radiology 290:691-699

3 Huang B, Wu L, Lu XY et al (2016) Small Intrahepatic Cholangiocarcinoma and Hepatocellular Carcinoma in Cirrhotic Livers May Share Similar Enhancement Patterns at Multiphase Dynamic MR Imaging. Radiology 281:150-157

4 Kim H, Park MS, Choi JY et al (2009) Can microvessel invasion of hepatocellular carcinoma be predicted by pre-operative MRI? Eur Radiol 19:1744-1751

5 Park HJ, Kim YK, Park MJ, Lee WJ (2013) Small intrahepatic mass-forming cholangiocarcinoma: target sign on diffusion-weighted imaging for differentiation from hepatocellular carcinoma. Abdom Imaging 38:793-801

6 Li P, Liang Y, Zeng B et al (2022) Preoperative prediction of intra-tumoral tertiary lymphoid structures based on CT in hepatocellular cancer. Eur J Radiol 151:110309

7 Yang Y, Zou X, Zhou W et al (2022) Multiparametric MRI-Based Radiomic Signature for Preoperative Evaluation of Overall Survival in Intrahepatic Cholangiocarcinoma After Partial Hepatectomy. J Magn Reson Imaging 56:739-751
