# Supplementary figures and images for: Using immunovascular characteristics to predict very early recurrence and prognosis of resectable intrahepatic cholangiocarcinoma
Source: BMC Cancer. 2023 Oct 19;23:1009. doi: 10.1186/s12885-023-11476-z (PMC10588260; doi:10.1186/s12885-023-11476-z)

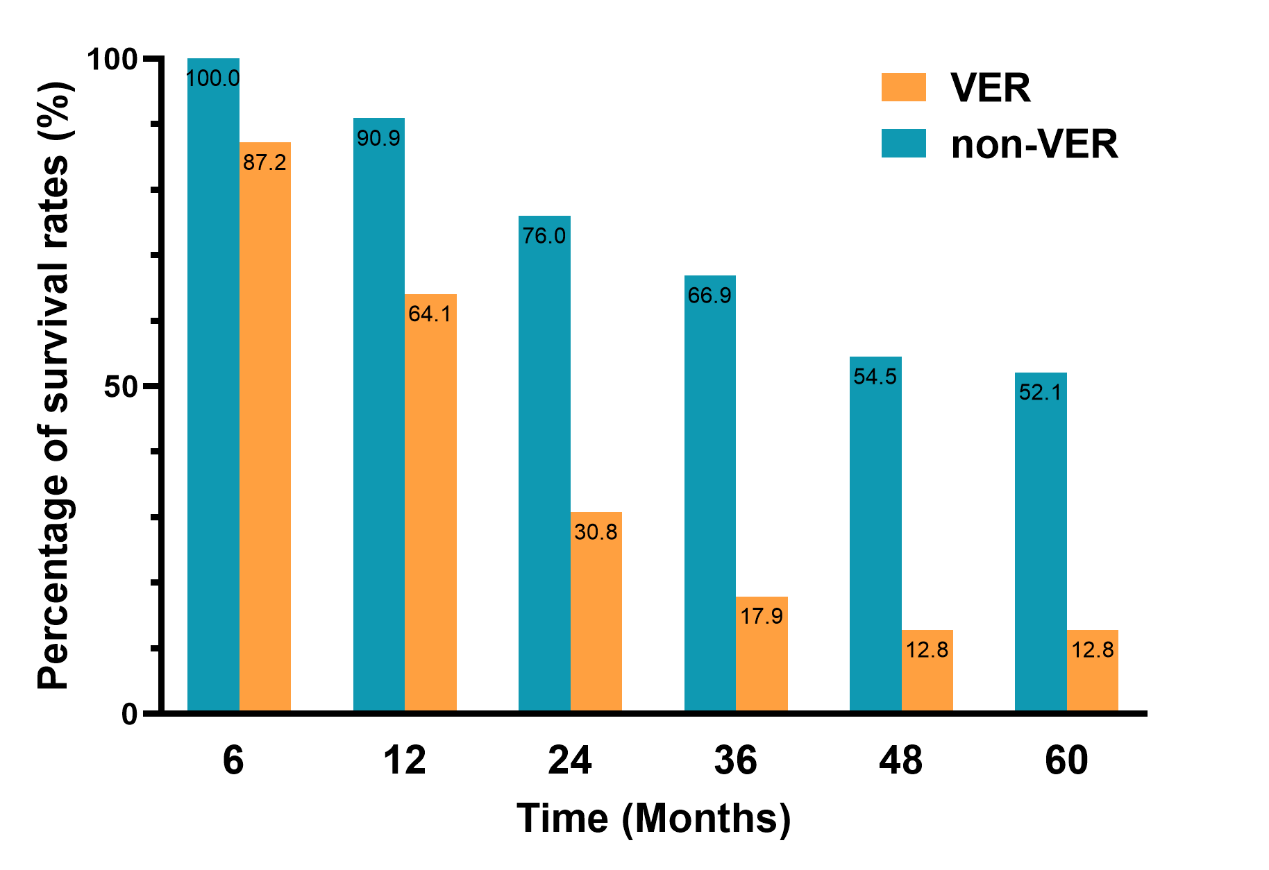


**S-Figure 1.** The bar graph of 6-, 12-, 24-, 36-, 48-, and 60-month survival rates of the VER and non-VER groups.

Supplement: Supplementary file 2 — Supplementary Material 2 [file 12885_2023_11476_MOESM2_ESM.docx]
